# Prognosis of bacteremic patients during postoperative peritonitis

**DOI:** 10.1186/2197-425X-3-S1-A120

**Published:** 2015-10-01

**Authors:** A Alqarni, P Augustin, A Snauwaert, E Guivarch, M Benrehouma, C Geneve, N Grall, K Arapis, P Montravers

**Affiliations:** Department of Anaesthesiology, CHU Bichat-Claude Bernard, Paris, France; Microbiology Laboratory, CHU Bichat-Claude Bernard, Paris, France; General Surgery, CHU Bichat-Claude Bernard, Paris, France

## Introduction

Blood stream infections of abdominal origin are associated with a high mortality rate. No data are available in patients (pts) with postoperative peritonitis (POP).

## Objectives

We hypothesized that both patient and microbiological factors determine outcome in critically ill POP pts.

## Methods

Between 1999 and 2014, all consecutive ICU pts admitted for management of POP were prospectively included in a data base. Bacteremic pts (B+) were defined as having at least one positive blood culture in the 24 hours preceding/following surgery for POP. The following data were collected: demographic characteristics, underlying disease, severity scores at the time of reoperation, microbiologic results, outcome and survival. B+ pts were compared to non-bacteremic pts (B-). Results are expressed in mean (SD) or proportions.

## Results

Overall, 292 pts (53% male, aged 60 (17) year old, 33% with fatal underlying disease) were enrolled, including 58 (20%) B+ pts. Age was similar in both groups while immunosuppression and cancer were more frequent in B+ pts (p < 0.001 and p < 0.02, respectively). No difference was observed for the characteristics of initial surgery leading to POP. Delay for reoperation (9 (8) days), location and cause of POP were similar in both groups. At the time of reoperation for POP, SAPS II score was similar in both groups (48 ± 18) while SOFA was increased in B+ pts (9 (4) versus 7 (4); p < 0.05). The organisms cultured from blood cultures (19% multidrug resistant bacteria (MDR), mainly Gram+ strains) were Gram+ (34%), Gram- (47%), anaerobes (14%) and fungi (10%). Among the organisms cultured from the peritoneal space, proportions of E. coli and Enterobacter spp were increased in B+ pts (55 vs 41% in B- pts; p < 0.05) and (28 vs 15%, p < 0.02), while the proportions of MDR bacteria were similar in both groups. Adequacy of empiric antibiotic therapy was reached in 69% of the cases, similar in both groups, without any difference in the type of agents used, frequency of combination therapy (75 and 80% in B+ and B- groups) or duration of therapy (11 (6) days). The frequency of de-escalation (51% in B+ vs 55% in B- pts) and escalation therapy (19% vs 25%, respectively) was similar in both groups. While death rate was increased in B+ pts (47% vs 31% in B- pts; p < 0.05), the morbidity criteria did not differ between B+ and B- pts: frequency of reoperations (47% in both groups), duration of mechanical ventilation in survivors (11 (11) days), duration of ICU stay in survivors (18 (12) vs 21 (19) days in B-pts). Positive cultures of enterococci in the peritoneal fluid could be factor of poor prognosis in case of bacteremia (74% death rate vs 32% without enterococci; p < 0.01).

## Conclusions

In our ICU population of POP, an increased death rate was reported in bacteremic pts but few elements explain this observation except underlying disease, ongoing cancer and possibly presence of enterococci within peritoneal samples.
